# An Open-Source 3D-Printed Hindlimb Stabilization Apparatus for Reliable Measurement of Stimulation-Evoked Ankle Flexion in Rat

**DOI:** 10.1523/ENEURO.0305-23.2023

**Published:** 2024-03-01

**Authors:** Danny V. Lam, Madeline Lindemann, Kevin Yang, Derrick X. Liu, Kip A. Ludwig, Andrew J. Shoffstall

**Affiliations:** ^1^Department of Biomedical Engineering, Case Western Reserve University, Cleveland 44106, Ohio; ^2^Advanced Platform Technology Center, Louis Stokes Cleveland Department of Veterans Affairs Medical Center, Cleveland 44106, Ohio; ^3^Department of Neurosurgery, University of Wisconsin-Madison, Madison 53705, Wisconsin

**Keywords:** electrodes, electromyography, peripheral nervous system, printing, three-dimensional, torque, transducers

## Abstract

Currently there are numerous methods to evaluate peripheral nerve stimulation interfaces in rats, with stimulation-evoked ankle torque being one of the most prominent. Commercial rat ankle torque measurement systems and custom one-off solutions have been published in the literature. However, commercial systems are proprietary and costly and do not allow for customization. One-off lab-built systems have required specialized machining expertise, and building plans have previously not been made easily accessible. Here, detailed building plans are provided for a low-cost, open-source, and basic ankle torque measurement system from which additional customization can be made. A hindlimb stabilization apparatus was developed to secure and stabilize a rat's hindlimb, while allowing for simultaneous ankle-isometric torque and lower limb muscle electromyography (EMG). The design was composed mainly of adjustable 3D-printed components to accommodate anatomical differences between rat hindlimbs. Additionally, construction and calibration procedures of the rat hindlimb stabilization apparatus were demonstrated in this study. In vivo torque measurements were reliably acquired and corresponded to increasing stimulation amplitudes. Furthermore, implanted leads used for intramuscular EMG recordings complemented torque measurements and were used as an additional functional measurement in evaluating the performance of a peripheral nerve stimulation interface. In conclusion, an open-source and noninvasive platform, made primarily with 3D-printed components, was constructed for reliable data acquisition of evoked motor activity in rat models. The purpose of this apparatus is to provide researchers a versatile system with adjustable components that can be tailored to meet user-defined experimental requirements when evaluating motor function of the rat hindlimbs.

## Significance Statement

The 3D-printed hindlimb stabilization apparatus provides a low-cost, open-source, and basic ankle torque measurement system from which additional customizations can be made. This will reduce the amount of time and energy spent by new researchers to replicate these methods in the fields of neural interface development, nerve injury study, and the study of therapies for other neuromuscular and neurodegenerative disorders.

## Introduction

The peripheral nervous system facilitates communication between the central nervous system and visceral organs, transmitting sensory, motor, and autonomic information. Peripheral neural interfaces (PNIs) provide tools to alter and/or record nerve activity and play a vital role in novel clinical therapies, diagnostics, and research. Various PNI electrodes exist on market, differing in stimulation and recording capabilities (Lago et al., 2007; Günter et al., 2019; Cho et al., 2020; Carnicer-Lombarte et al., 2021). Despite recent PNI adoptions for therapeutic nerve stimulation, improved electrode designs are necessary to reduce patients’ risks and improve clinical outcomes, including device migrations, lead fractures and breakages, or unintended infections (Lambru and Matharu, 2012; Eldabe et al., 2016). However, evaluation of new PNI designs is limited due to the lack of standardized metrics where implementation techniques vary in invasiveness and measurement types.

Evoked motor function can be directly assessed through force measurements from surgically isolated muscles, but cannot be used for chronic evaluation due to disruptions to physiological processes (MacIntosh et al., 2011; Santocildes et al., 2022). Intramuscular electromyography (EMG) is less invasive, where electrical activity is recorded from electrodes inserted into target muscles. However, this method is susceptible to noise contamination and stimulation artifacts (English et al., 2007). Moreover, repeated insertions cause tissue trauma and scar formation that may hinder future measurements (Akers et al., 1997; Burnham et al., 2006). Applications with implanted EMG leads face similar challenges where a sustained foreign body response impacts the signal quality over time (Grill et al., 2009; Gao et al., 2022). Surface EMG recordings offer a noninvasive approach but faces further challenges in recorded signal quality (De Luca et al., 2010; Qiu et al., 2015).

Video-based analyses offer a noninvasive method to evaluate motor function over time without perturbations to target muscles. Spatial and temporal tracking of position and orientation of body segments can be implemented to measure kinematic changes (Amado et al., 2011; Abbas and Masip Rodo, 2019). However, reliance on user-defined landmarks can lead to inconsistencies in data quality. For example, many video-capture systems require consistent alignment of identified landmarks for accurate data capture and representation.

Ankle torque is a published noninvasive method for measuring muscle contractility (Warren et al., 1998; Iyer et al., 2016). Ankle torque measurements can be used to evaluate the chronic performance of PNIs as a functional outcome and may provide indirect measurements of potential lead failures, such as undesirable lead migration. Current literature on stabilizing the hindlimb for consistent ankle joint measurements either lacks comprehensive design guidelines or features custom-made components with insufficient design and construction information (Grill and Mortimer, 1996; Stieglitz et al., 2003; Ichihara et al., 2009).

Here, a customizable open-source hindlimb stabilization apparatus was constructed primarily of 3D-printed components and secures the rat's knee and foot. A torque transducer was equipped to a foot pedal, allowing for repeated collection of evoked ankle flexion. Compared with other systems, the proposed hindlimb stabilization apparatus offers a highly customizable and cost-effective solution, making it ideal for laboratories evaluating PNIs.

## Material and Methods

### Hindlimb stabilization apparatus design, manufacturing, and implementation

**Figure 1.**
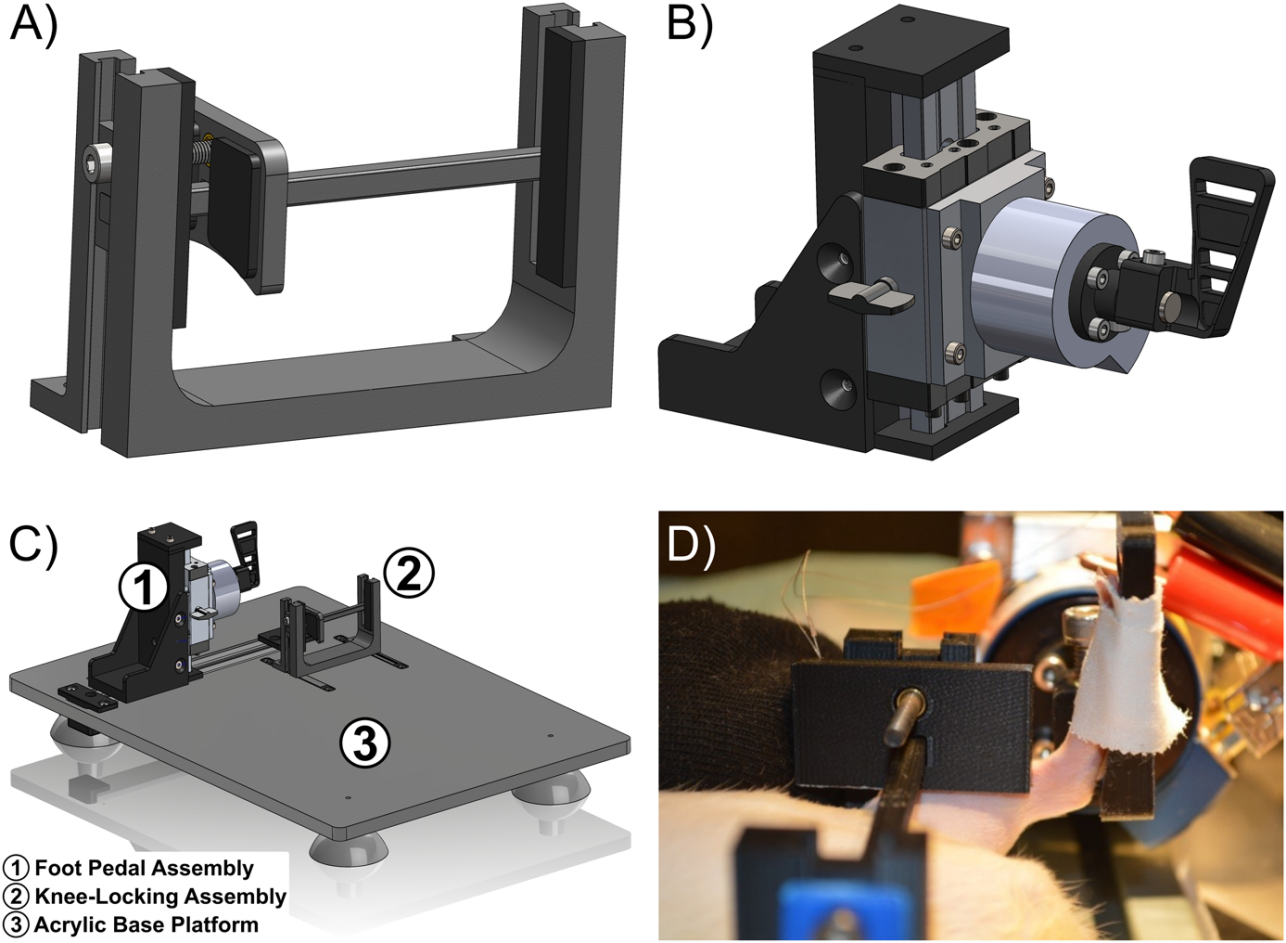
Overview of individual components within the hindlimb stabilization apparatus. Measurements from individual rats (*n* = 9) were obtained to optimize dimensions of individual components (Table 1-1). Printing specifications for individual components (Table 1-2) and bill of materials for additional hardware (Table 1-3) were provided for construction of the entire apparatus. ***A***, The knee-locking assembly was mainly composed of 3D-printed components with screws that allow for adjustments and positioning of the hindlimb. ***B***, Foot pedal assembly was composed of a combination of machined and 3D-printed components with screws that allow for adjustments and positioning mounted torque transducer. ***C***, General schematic of the mounted knee-locking and foot pedal assemblies onto an acrylic base platform. ***D***, Example of a rat hindlimb secured into both the knee-locking and foot pedal assemblies. Additional perspectives of proper hindlimb placement and securement were demonstrated (Fig. 1-1).

The overall hindlimb stabilization apparatus was designed to accommodate anatomical differences of the hindlimbs for adult male and female rats. Rat carcasses (*n* = 9, Sprague Dawley) of varying weights (250–450 g) were used as anatomical references to design the knee-locking and foot pedal assemblies. The anatomical dimensions were measured for rats’ full body, hindlimb, and foot (Table 1-1). The knee-locking (Figs. 1*A*, 3*A*) and foot pedal assemblies (Figs. 1*B*, 4*A*) were mounted onto an acrylic base platform containing sliding rails. Sliding rails enable horizontal adjustments of the knee-locking assembly by up to 95 mm and the foot pedal assembly by up to 125 mm. The knee-locking assembly incorporates vertical posts that allow for height adjustments by up to 65.5 mm while the foot pedal assembly uses a sleeve-bearing carriage attached with a guide rail for height adjustments by up to 87.5 mm. After adjusting each component, tightening screws were used to secure each component in their respective positions for each assembly. Individual components for respective assemblies were primarily 3D-printed under fused deposition modeling with polylactic acid (PLA) via Ultimaker 3+ (Ultimaker B.V.) of variable infill settings (Table 1-2). Additional hardware and machined components for design implementation were included as a bill of materials (Table 1-3). CAD files for all parts and assemblies used were generated in SolidWorks (Dassault Systèmes SolidWorks Corporation) and can be accessed (https://github.com/Shofflab/Open-Source_Stabilization_Apparatus.git). After obtaining all the necessary components for this design, the hindlimb stabilization apparatus can be completely assembled, as shown in the schematic illustration (Fig. 1*C*). A rat's hindlimb was secured into the knee-locking and foot pedal assemblies to demonstrate appropriate positioning within the stabilization apparatus (Fig. 1*D*). Additional images from various perspectives were captured to illustrate proper placement and securement of the rat's hindlimb (Fig. 1-1).

10.1523/ENEURO.0305-23.2023.t1-1 Table 1-1 **Measurements from individual rat carcasses used in designing the hindlimb stabilization apparatus.** * = Measurement was not taken for this subject. Download Table 1-1, DOC file.

10.1523/ENEURO.0305-23.2023.t1-2 Table 1-2 **Printing specifications for 3D-printed components**. Infill (%), print time (hours, minutes), and unit mass (g) were calculated using the Ultimaker Cura software. All components were printed using PLA. * = Unit price calculated at $0.15 (USD) per gram. Download Table 1-2, DOC file.

10.1523/ENEURO.0305-23.2023.t1-3 Table 1-3 **Bill of materials for additional hardware used in design implementation**. Cost is shown at time of lookup (circa 2021). * = Unit cost covers the quantity required. Download Table 1-3, DOC file.

10.1523/ENEURO.0305-23.2023.f1-1 Figure 1-1 **Example placement of the rat's left hindlimb.** A, B, C) Additional views were provided to illustrate proper securement of the hindlimb after proper adjustment of the knee-locking and foot pedal assemblies. Download Figure 1-1, TIF file.

#### Mounting platform for assemblies

**Figure 2.**
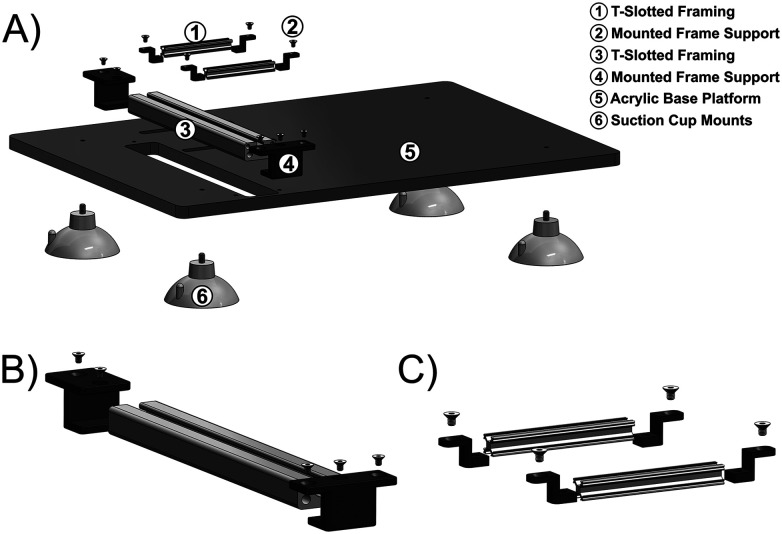
Schematic demonstration of the mounting platform. ***A***, Schematic of required parts to mount the knee-locking and foot pedal assemblies onto the acrylic base platform. ***B***, *t*-slotted framing with mounting supports was used to establish a railing design, enabling horizontal adjustments of the foot pedal assembly. ***C***, Two bars of miniature *t*-slotted framing with mounting supports were used to establish a railing design, enabling horizontal adjustments of the knee-locking assembly.

The mounting platform was designed to facilitate the positioning of a rat on a heating pad, featuring adjustable components that can be modified vertically and horizontally to accommodate rats with varying hindlimb sizes. The mounting platform consists of an acrylic base with dimensions of 540 mm × 380 mm (length, width) mounted on four threaded rubber suction cup mounts (McMaster-Carr) with a diameter of 79 mm. Rubber suction cup mounts were incorporated into the design to dampen displacement of the mounting platform during evoked muscle contraction. A CNC router was used to mill and drill the necessary geometry into the acrylic base. The acrylic base holds multiple adjustable components including lightweight aluminum sliding rails composed of *t*-slotted framing (McMaster-Carr) with 3D-printed supports (Fig. 2*A*). A single bar of *t*-slotted framing with dimensions of 253 mm × 40 mm × 20 mm (length, width, thickness) allowed horizontal movement of the foot pedal assembly along the sliding rail, allowing for a maximum displacement of 125 mm from the furthest edge of the foot pedal assembly (Fig. 2*B*). This dynamic range of the system permits torque measurement from both hindlimbs of the rat without further manipulation of other components. Two bars of miniature *t*-slotted framing with dimensions of 82 mm × 10 mm × 10 mm (length, width, thickness) allowed horizontal movement of the knee-locking assembly along the sliding rail within a range of 27.5–95 mm (Fig. 2*C*). Final lengths for the bars of *t*-slotted framing were all cut using a vertical band.

#### Knee-locking assembly

**Figure 3.**
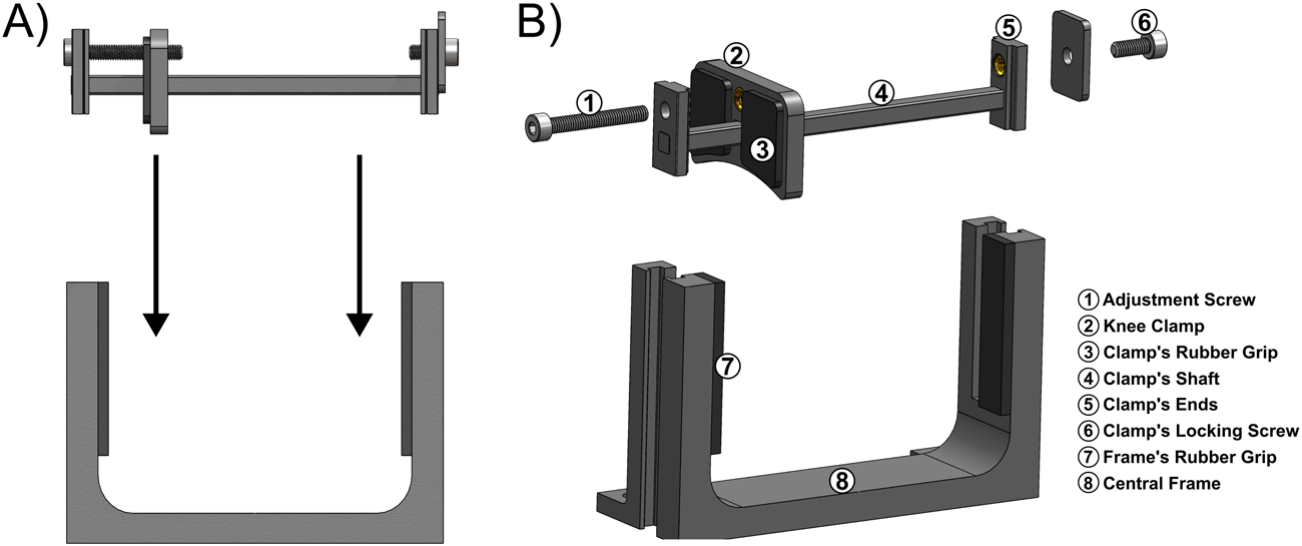
Schematic demonstration of the knee-locking assembly. ***A***, Placement of the knee-clamp shaft into the vertical posts of the central frame. ***B***, Individual components listed for the knee-locking assembly.

The knee-locking assembly includes a central frame with vertical posts, providing stability to the rat's body when positioned supine. An adjoining knee-clamp shaft was provided to secure the knee firmly in place. Both components were manufactured through 3D-printing using PLA filament. The central frame was specifically designed with horizontal adjustability to accommodate the rat's body along the sliding rails. It is securely fastened to the rails using button head cap screws. The ends of the knee-clamp shaft are placed into the openings of the central frame's vertical posts, allowing height adjustments of the knee-clamp shaft for a range of 25.7–65.5 mm (Fig. 3*A*). After determining the desired height, the knee-clamp shaft is firmly fastened to the central frame's post using a socket screw to ensure stability. Adhesive rubber paddings (McMaster-Carr) were implemented into the knee-locking assembly to reduce friction and minimize limb movement in regions where the rat's skin comes into contact. The knee-clamp can be horizontally adjusted to pin the knee against the vertical posts of the central frame (Fig. 3*B*). A downward concavity design was implemented into the knee clamp to contour the rat's hindlimb and maximum surface area contact. The knee-clamp shaft can be rotated 180° to accommodate torque measurements of the opposite hindlimb.

#### Foot pedal assembly

**Figure 4.**
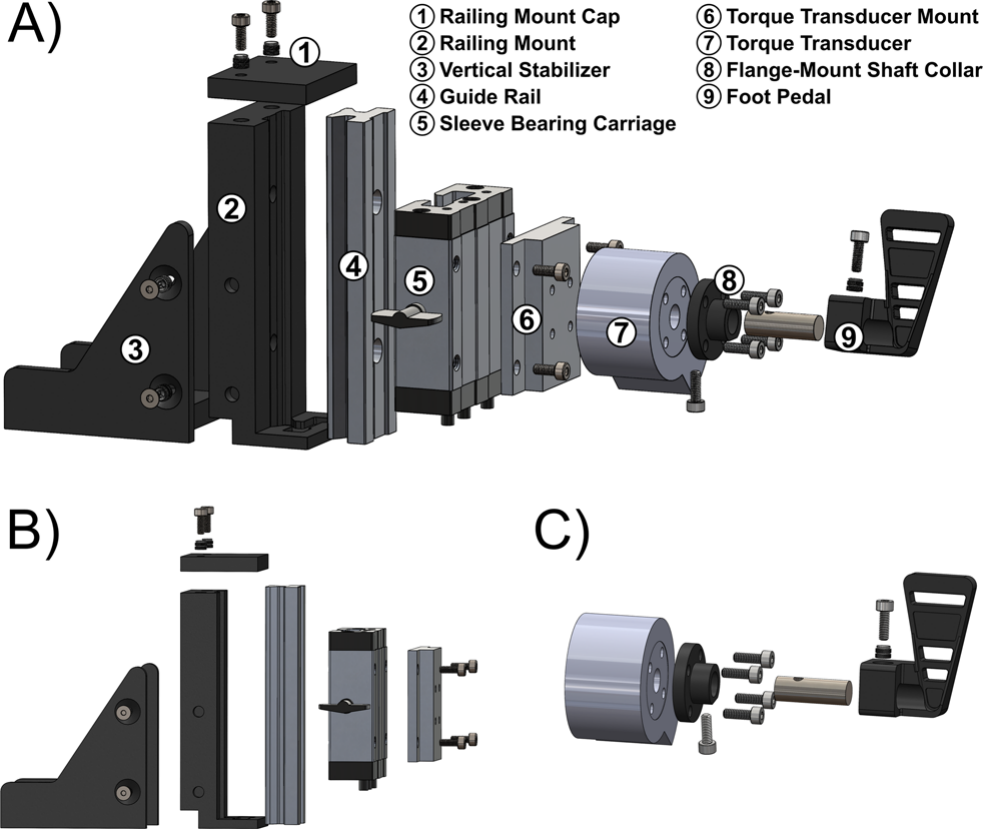
Schematic demonstration of the foot pedal assembly. ***A***, Exploded view of the foot pedal design with listed individual components. ***B***, Closer inspection of the mounting design that enables vertical and horizontal adjustments to the foot pedal assembly. ***C***, Closer inspection of the foot pedal–torque transducer design. Dimensions for specific components were provided for the transducer's stainless-steel shaft (Fig. 4-1), the flange-mounted shaft collar (Fig. 4-2), and the transducer's mount to the sleeve bearing carriage (Fig. 4-3).

The foot pedal assembly contains multiple components that are fabricated through 3D-printing and machining to optimize customizability for user-defined experiments (Fig. 4*A*). The foot pedal was 3D-printed using PLA filament with final dimensions of 57.8 mm × 22.7 mm (length, width with respect to ball of the foot; Fig. 4*B*). Final dimensions were referenced to previous rat foot measurements (Table 1-1). A triangular design for the foot pedal was implemented to enable toe spreading, optimizing the foot contact surface area. Open slots allow for visual inspection of foot alignment to the pedal before securement with tape wrapped around the midfoot and pedal. The right side of the foot pedal features an extruded surface to accommodate a 304 stainless-steel shaft with dimensions of 30 mm × 10 mm (length, diameter; McMaster-Carr) and serves to connect and align the torque transducer. The final dimensions of the shaft were selected to minimize shaft twisting when a load is applied to the foot pedal. A vertical band saw was used to cut the bulk stainless-steel shaft to ensure enough length to connect the foot pedal to the flange-mounted shaft collar of the torque transducer. The alignment of the shaft to the foot pedal was established such that the central axis of the torque transducer is colinear with the axis of the ankle joint. The center of ankle rotation was determined by identifying the center of the rat foot (Table 1-1). A set screw is threaded through the foot pedal using a brass insert with matching threads and allows for the securement of the shaft and angle adjustments to the foot pedal. This configuration allows the operator to find the optimal angle for isometric muscle contraction during use, preferably at a neutral position such as 90° (Yamauchi and Koyama, 2019). At one end of the shaft, a threaded hole was machined and tapped through the central cross section to facilitate the connection between the shaft and the flange-mounted shaft collar (Fig. 4-1). The flange-mounted shaft collar was customized to ensure proper coupling of the foot pedal's shaft to the torque transducer (Fig. 4-2). The flange-mounted shaft collar was 3D-printed using PLA filament and was attached to the torque transducer with socket head screws.

10.1523/ENEURO.0305-23.2023.f4-1 Figure 4-1 **Dimensions (inches) for the cut stainless-steel shaft used to connect and align the torque transducer to the foot pedal.** Download Figure 4-1, TIF file.

10.1523/ENEURO.0305-23.2023.f4-2 Figure 4-2 **Dimensions (inches) for flanged-mount shaft collar used to couple the foot pedal's shaft to the torque transducer.** Download Figure 4-2, TIF file.

10.1523/ENEURO.0305-23.2023.f4-3 Figure 4-3 **Dimensions (inches) for machined mount used for the torque transducer in the foot pedal assembly.** Download Figure 4-3, TIF file.

A strain gauge transducer (model 20E12, JR3) can measure force and torque along three axes, providing more data for evaluating motor responses. The torque data collection was solely concentrated on the *z*-axis for demonstration purposes (Specification Sheets - JR3 Inc.). The torque transducer comes with a DE-19 connector for signal acquisition and front-end interfacing, including signal conditioning, amplification, and filtering. It has a nominal range of 3 N-m and a sensitivity of 0.39 N-mm with respect to the *z*-axis. To mount the torque transducer, we fabricated a customized component from 6,061 aluminum stock (McMaster-Carr). The fabrication process involved cutting the aluminum stock using a vertical bandsaw and machining it to its final specifications using a mill (Fig. 4-3). The torque transducer mount is fixed onto a sleeve-bearing carriage attached with a guide rail (McMaster-Carr; Fig. 4*C*). This configuration allows for height adjustment of the foot pedal within a range of 48–87 mm. The sleeve-bearing carriage is equipped with a handbrake to secure the foot pedal and its attached accessories into position. A railing mount secures the guide rail in position. In addition, a vertical stabilizer was added to this design to stabilize the railing mount and serves as the base attachment for the foot pedal assembly to the acrylic base railing. The base attachment is secured to the railing mount with four flathead screws, enabling the detachment of the railing mount from the acrylic base without removing the acrylic base railing. The decision to design the railing mount and base attachment as distinct components serves to distribute the load of the foot pedal assembly evenly on the railing mount. This distribution occurs across the fasteners that connect these components, intentionally placed in areas of high-stress concentration. A railing mount cap was included in this design to safeguard against sharp edges and enhance the stability of the guide rail and railing mount.

### Transducer calibration for torque measurements

**Figure 5.**
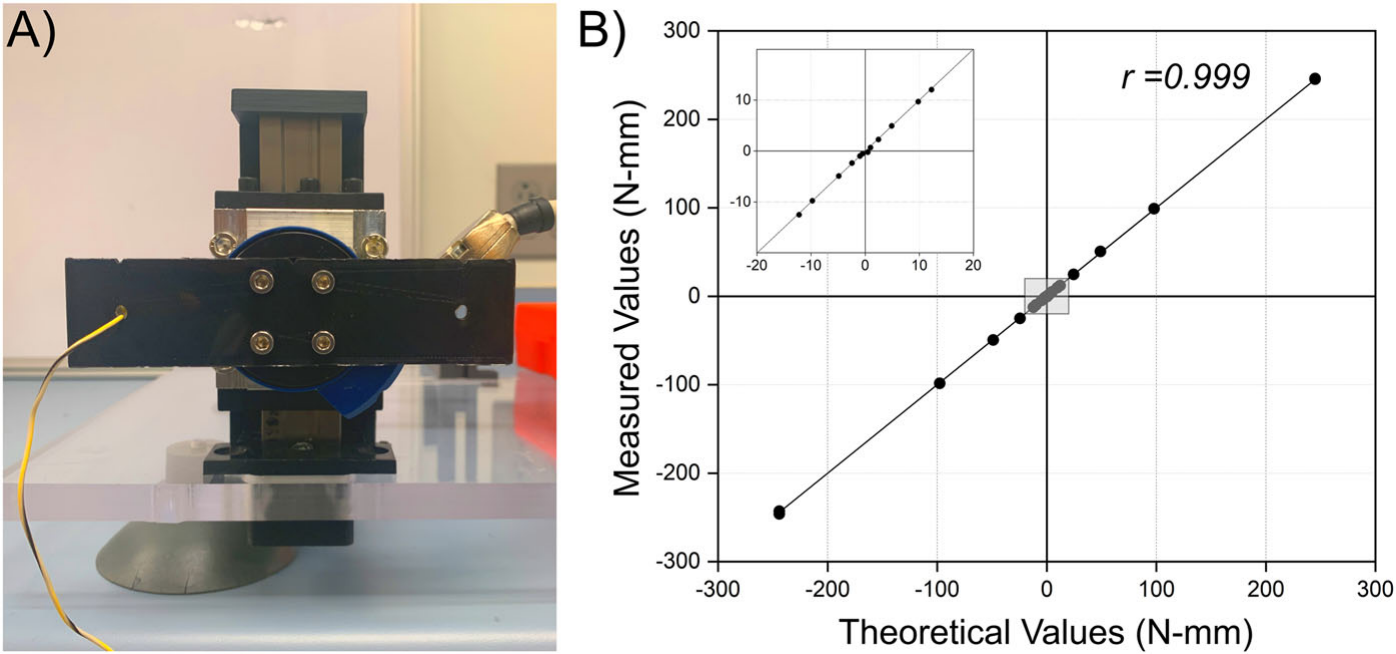
Calibration setup for data acquisition of torque about a single axis. ***A***, Mounted cantilever used during calibration of the torque transducer. Dimensions for the cantilever used in this study were provided (Fig. 5-1). ***B***, The plotted torque values in comparison with the theoretical values exhibited a robust correlation, indicating a high level of confidence in the accuracy of the measurements obtained during calibration (*r* = 0.999; *p* < 0.005).

Calibrated weights (Ohaus) of known masses were suspended from the end of the cantilever using a thread passed through the side mounting holes of the transducer (Fig. 5*A*). The cantilever used in this study was 3D-printed from PLA filament with dimensions of 130.4 mm × 31.5 mm × 5 mm (length, width, thickness; Fig. 5-1). The 3D-printed cantilever's moment arms were measured as 49.7 mm and 49.9 mm from the cantilever ends to the center, respectively. Theoretical torque measurements were calculated as a product of the calibrated weights, with respect to gravity, suspended at either ends of the cantilever. Data collection was initiated 10–15 s after weight suspension to limit possible swinging of the suspended weights. A total of 1,500 samples of torque measurements were digitized at 10 Hz via a DE19-P connector (JR3) to a 16 bit analog-to-digital converter (NI-DAQ model 9222, National Instruments). Baseline torque was determined for no attached weights and subtracted from recorded torque measurements for each calibrated weight. The average torque measurements were recorded for both the left and right sides of the cantilever to represent both positive and negative torques around the *z*-axis (Table 1).

10.1523/ENEURO.0305-23.2023.f5-1 Figure 5-1 Cantilever dimensions (inches) for calibration of the torque transducer. Download Figure 5-1, TIF file.

**Table 1.** Error between corrected measured and theoretical torque values per calibrated weight

Calibrated weight (g)	Positive torque about the *z*-axis			Negative torque about the *z*-axis		
	Measured value (N-mm)	Theoretical value (N-mm)	Error (%)	Measured value (N-mm)	Theoretical value (N-mm)	Error (%)
500	245.3	245.0	0.14	−243.1	−244.1	−0.43
200	99.1	98.0	1.07	−98.5	−97.6	0.85
100	50.7	49.0	3.36	−49.4	−48.8	1.21
50	24.6	24.5	0.55	−25.0	−24.4	2.20
25	12.0	12.2	−1.67	−12.5	−12.2	2.25
20	9.70	9.80	−1.05	−9.74	−9.76	−0.20
10	4.94	4.90	0.79	−4.89	−4.88	0.09
5	2.28	2.45	−7.52	−2.36	−2.44	−3.48
2	0.69	0.98	−41.7	−0.98	−0.98	0.00
1	−0.22	0.49	322.3	−0.52	−0.49	5.25

### Intra-operator variability

To evaluate the data collection reliability of the hindlimb stabilization apparatus, we categorized three operators as “beginner,” “intermediate,” and “expert” based on their experience in animal handling and familiarity with the apparatus. Operators were instructed on correct positioning of the rat's hindlimb in the knee-locking and foot pedal assemblies, ensuring proper alignment and securement of each leg part. The hindlimb was repositioned before each data collection. Each operator performed three separate trials of torque measurements on each subject (*n* = 3) at a stimulation amplitude of 1.0 mA for each hindlimb, resulting in a total of 18 measurements. To accommodate for individual animal variability, the recorded torque measurements were normalized relative to the average torque of the limb from which they were obtained.

### Animal use and surgical procedures

All animal procedures were performed in accordance with the Case Western Reserve University's animal care committee's regulations. Male Sprague Dawley rats (225–250 g, Charles River Laboratories) were used and housed for up to 2 weeks before surgery. Anesthesia was induced at 3–4% isoflurane and maintained at 1.5–2% during surgery. Subjects used in this study were killed via carbon dioxide after acute surgeries and data collection.

Rat sciatic nerve stimulation (*n* = 3) was conducted to assess hindlimb stabilization and repeatable collection of torque measurement. Prior to surgery, anesthetized subjects were positioned onto the acrylic platform of the hindlimb stabilization apparatus containing appropriate surgical drapes and a warming pad (CODA, Kent Scientific Corporation). In brief, a 2–3 mm midline skin incision was made lateral to the femur, followed by blunt separation of vastus lateralis and bicep femoris muscles. The 316LVM stainless-steel wires (4 mm diameter fluoropolymer insulated, Fort Wayne Metals) were fashioned into “hook electrodes” and sutured to the epineurium of the sciatic nerve and the surrounding muscle plane to minimize movement of the wire during surgery. The skin incision was sutured closed with the ends of the uninsulated wires extending out of the incision. Subjects were positioned into the central frame of the knee-locking apparatus. The knee-clamp shaft was lowered toward the subject's hips and positioned onto the subject's anterior thigh. The knee was raised to a 90° angle with respect to the hip and secured in position by securing the knee-clamp to the vertical posts of the central frame. To prevent further movement of the lower body, the knee-clamp shaft was secured to the vertical posts of the central frame. The subject's foot was then secured to the foot pedal at a neutral position, 90° angle with respect to the lower leg. Vertical and horizontal adjustments of individual components for both the knee-locking and foot pedal assemblies can be achieved to accommodate anatomical differences. The wire ends that were exposed following closure of the skin incisions were connected to the input and ground terminals of an AC/DC current source (model 6221, Keithley Instruments) for electrical stimulation. Surgical procedures were repeated for the contralateral hindlimb.

A separate subject was used to demonstrate the feasibility and functional measurements of a PNI in development. Surgical procedures were conducted similarly as previously mentioned. After sciatic nerve exposure, coiled gold microwires (Neuronoff) were implanted under the sciatic nerve and into the belly of the gastrocnemius lateralis muscle. In addition to torque measurements, evoked intramuscular EMG was evaluated as an additional feature of the hindlimb stabilization apparatus. Stainless-steel wires (Cooner Wire) served as reference and ground wires. The reference wire was placed in nearby adipose tissue of the respective hindlimb. The ground wire was placed in adipose tissue of the back, ∼5 cm proximal to reference wires. Uninsulated wire ends were extended out of the sutured skin incisions for electrical stimulation and intramuscular EMG recording. Subjects were positioned into the hindlimb stabilization apparatus prior to sciatic nerve stimulation for ankle torque measurements.

### Sciatic nerve stimulation

Charged balanced biphasic pulses (cathodic-leading) were delivered to the sciatic nerve in a monopolar configuration. Stimulation amplitudes were randomized for each consecutive pulse train to limit muscle fatigue at a step size of 0.1 mA. Other stimulation parameters include 10 Hz frequency, 100 μs pulse width, and 25 μs inter-pulse delay for a 1 s stimulation train.

### Evoked torque and intramuscular electromyography measurements

Evoked torque measurements were digitized at 10 Hz via a DE19-P connector (JR3) to a 16 bit analog-to-digital converter (NI-DAQ model 9222, National Instruments). Torque measurements were detrended to remove baseline noise. Peak torque was calculated as absolute values for comparisons between plantar- and dorsi-flexion responses. Intramuscular EMG signals were digitized at ∼25 kHz sampling rate through the Lab Rat recording system (Tucker-Davis Technologies) and digital filtered (bandpass, 1–200 Hz; notch, 60, 120, and 180 Hz) through the complementary software, Synapse Lite. A custom Python script (version 3.7) was written for EMG quantification. Stimulation artifacts were detected using peak thresholds for each pulse within a given stimulation train. EMG waveforms were quantified in an 8 ms window after the stimulation artifact and reported as root mean square (RMS).

### Statistics

A custom Python script (version 3.7) was used to perform all statistical analysis. A Pearson's correlation was evaluated for linear relationships between variables such as torque measurements and stimulation amplitudes (*p* < 0.005). Sigmoid curves were fitted to evaluate nonlinear relationships between variables of interest, such as establishing dose–response curves (*p* < 0.005). A Shapiro–Wilk test for normality (*p* < 0.005) was performed on recorded torque measurements from operators (beginner, intermediate, and expert). A one-way ANOVA was conducted to evaluate differences in torque measurements between operators with different experience levels using the stabilization apparatus (*p* < 0.005). Additionally, a one-way ANOVA, followed by Tukey’s HSD test were conducted to evaluate differences for evoked intramuscular EMG signals and torque measurements (*p* < 0.005).

## Results

### Device calibration

Raw torque values about a single axis from known weights were recorded from a suspended cantilever attached to the torque transducer (Fig. 5*A*). The conversion factor was determined as 0.49 N-mm/g based on the dimensions of the cantilever used in this study. Measured torque values were greater than theoretical values by a magnitude of 3.49 ± 0.08 for either positive or negative torque (Table 1). The final calibration excluded values for given weights of 1 or 2 g as theoretical values were approaching the torque transducer's sensitivity limitations. The error rate between the corrected measured values and theoretical values was calculated, and minimal differences were observed between the measured and corresponding theoretical values for most weights (Table 1). However, larger errors accumulated around the sensitivity limitations of the torque transducer (0.39 N-mm; JR3). Torque measurements at the negative direction produced the least error rates at weights <25 g. Pearson’s correlation coefficient was calculated to assess the linear relationship between the measured and theoretical values, where a strong positive correlation existed (*r* = 0.999; *p* < 0.005; Fig. 5*B*).

### Data acquisition of torque measurements during rat sciatic nerve stimulation

**Figure 6.**
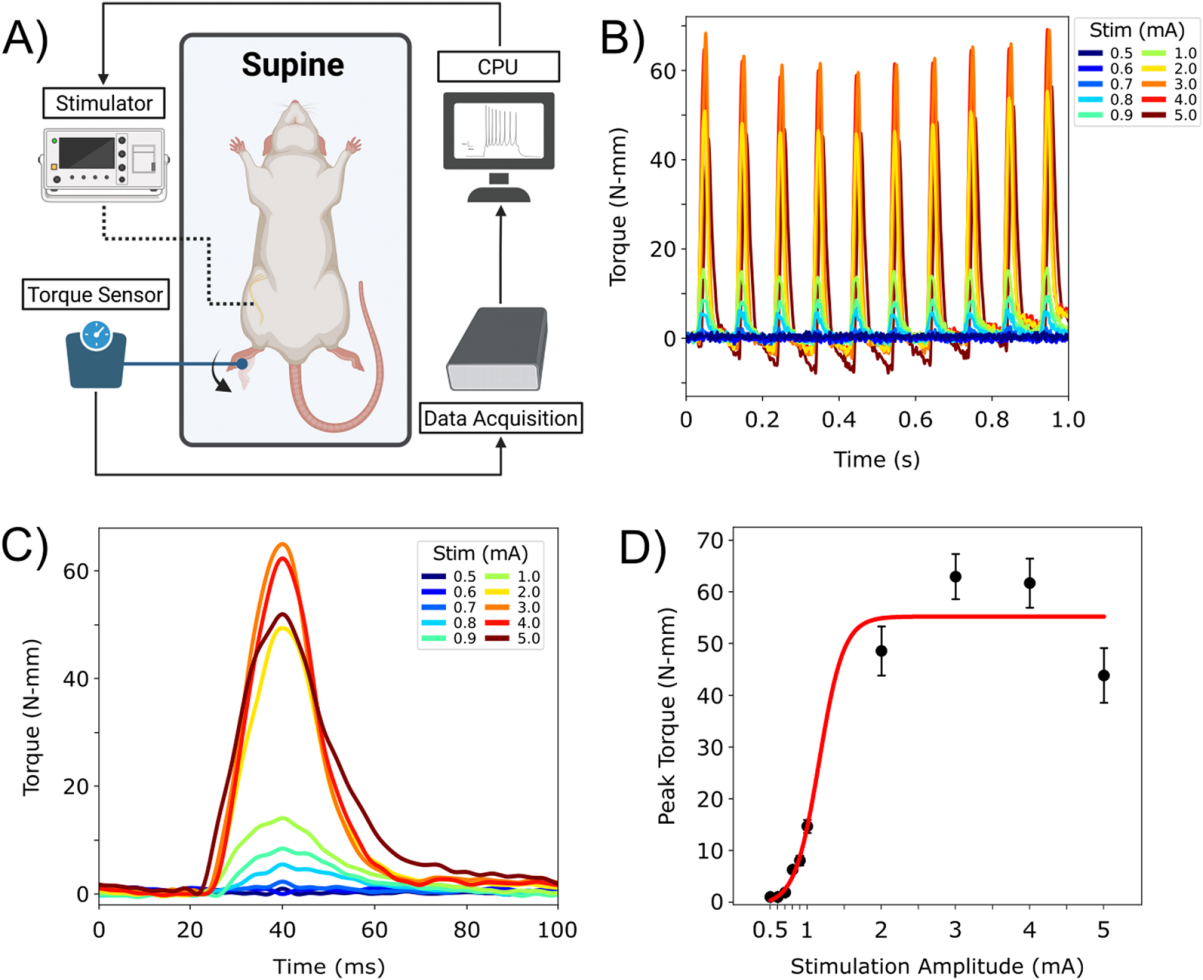
Demonstration of recorded torque measurement from a representative subject (*n* = 1) during sciatic nerve stimulation. ***A***, A workflow schematic illustrates the process of data acquisition. Prior to placement into the hindlimb stabilization apparatus, subjects were positioned supine. Electrical nerve stimulation was initiated by programming stimulation parameters into the current source. Simultaneously, evoked ankle flexion of the hindpaw was recorded from the torque transducer during sciatic nerve stimulation as either dorsi- or plantar-flexion (Fig. 6-1). ***B***, Example raw torque measurements were aligned and plotted for stimulation amplitudes within a range of 0.5–5.0 mA. ***C***, The mean torque waveform for each stimulation amplitude from respective stimulation trains was extracted, aligned, and plotted. ***D***, Peak torque was calculated for each respective stimulation amplitudes to produce a dose–response curve, shown as mean ± SD (*r* = 0.953; *p* < 0.005).

After confirming the reliability of the hindlimb stabilization apparatus for data collection, in vivo recordings of evoked torque measurements were acquired. A schematic was provided to demonstrate the experimental procedure for sciatic nerve stimulation and data collection (Fig. 6*A*). In summary, subjects were positioned supine over a heating pad to maintain their body temperature. A desktop computer was utilized to interface with the current source, allowing for user–input stimulation parameters used for electrical stimulation. During sciatic nerve stimulation, evoked ankle torque was observed as either plantar- or dorsi-flexion (Fig. 6-1). To further demonstrate data acquisition, example torque measurements from a representative subject (*n* = 1) were obtained at stimulation amplitudes within a range of 0.5–5.0 mA (Fig. 6*B*). Torque responses evoked by stimulation were observed to increase with respect to stimulation amplitudes. To further illustrate this relationship, the mean torque waveform was calculated for each stimulation train (10 samples for a 1 s time window) at respective stimulation amplitudes (Fig. 6*C*). A strong relationship between evoked ankle torque with respect to the stimulation amplitudes was observed within the dose–response curve from a range of 0.5–5.0 mA (*r* = 0.953; *p* < 0.005; Fig. 6*D*). Peak torque for respective stimulation amplitudes were calculated and exhibited minimal torque variability. The maximum peak torque, 65.0 ± 1.67 N-mm, was observed at a stimulation amplitude of 3.0 mA. The peak torque was observed to decrease after 3.0 mA, recording a response of 52.0 ± 2.66 N-mm for a stimulation amplitude of 5.0 mA. This decline in peak torque may be explained by activation of both plantar- and dorsi-flexion that leads to a twitch-like response, as observed by a negative dip following the positive peak response during 5.0 mA stimulation (Fig. 6*B*).

10.1523/ENEURO.0305-23.2023.f6-1 Figure 6-1 **Example of recorded torque measurements for A) plantar- and B) dorsi-flexion.** Respective ankle flexions were evoked with the following stimulation parameters: 1.0mA, 10Hz frequency, 100μs pulse width, and 25μs inter-pulse delay. Download Figure 6-1, TIF file.

**Figure 7.**
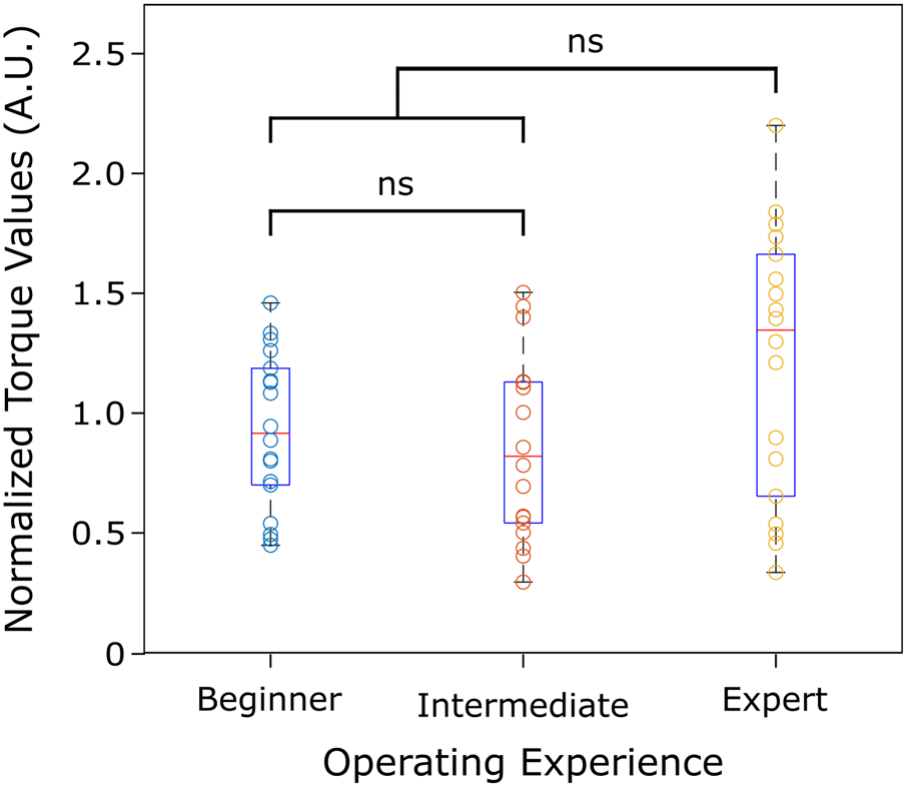
Collection of torque measurements was consistent regardless of the operator's user experience. No significant differences (ns) were detected with respect to the users’ operating experience in using the hindlimb stabilization apparatus (*p* < 0.005). Individual normalized torque values for each operator were recorded (Table 7-1). Red lines, blue boxes, and black whiskers represent medians, interquartile ranges, and maximum and minimum values, respectively.

Three operators of varying experience in using the proposed apparatus collected triplicate trials of torque measurements from each hindlimb (left and right) at a stimulation amplitude of 1.0 mA. To address inter- and intra-animal variability, torque measurements from each hindlimb were normalized according to the respective limb from which they were obtained. The range of acquired torque values was consistent across different levels of operating experience (*p* < 0.005). Consistent evoked torque measurements were achievable with proper hindlimb positioning and securement, regardless of the operator's familiarity with the apparatus (Fig. 7).

10.1523/ENEURO.0305-23.2023.t7-1 Table 7-1 Individual normalized torque recordings collected by respective operators. Download Table 7-1, DOC file.

### Comparison of functional measurements of evoked torque and intramuscular EMG recordings

**Figure 8.**
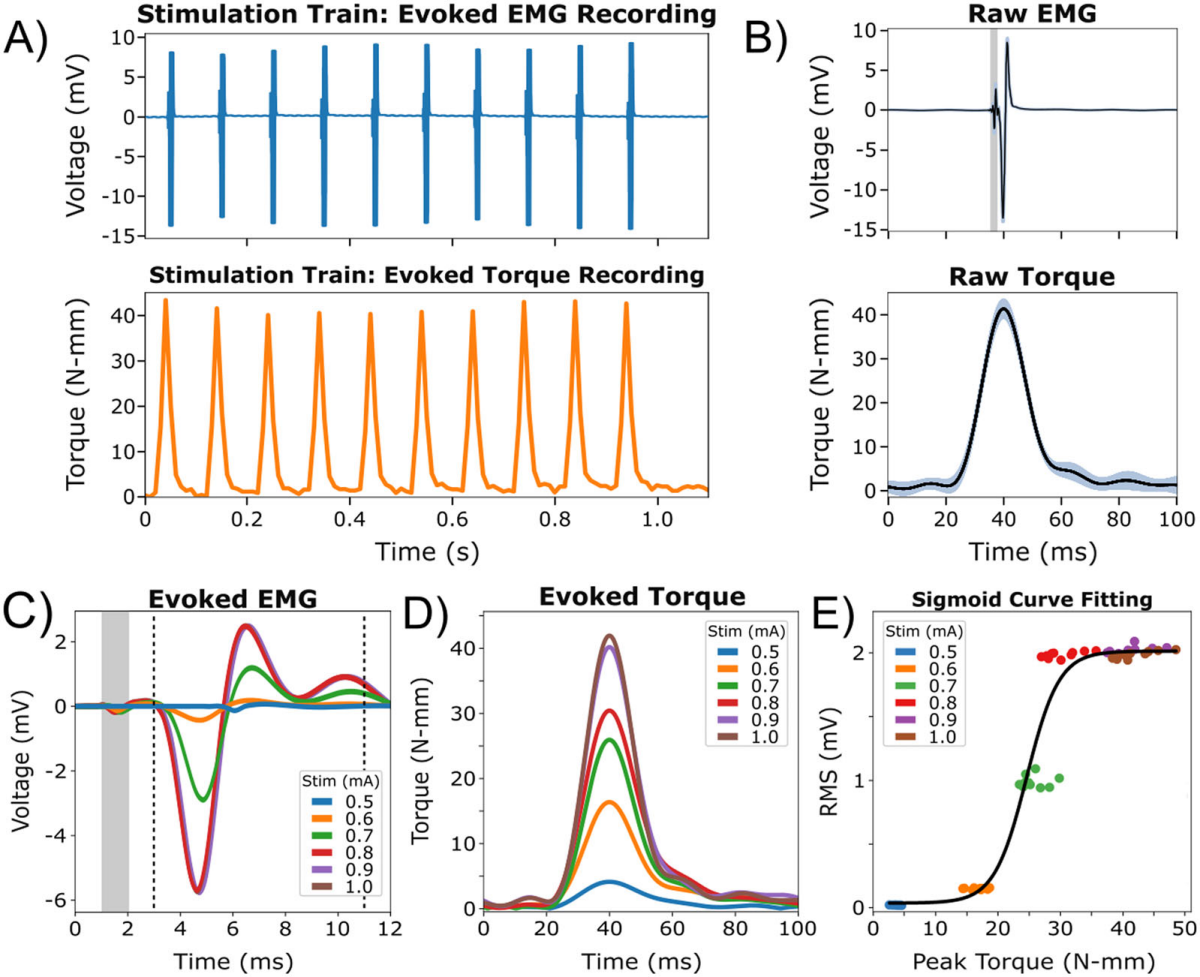
Intramuscular EMG recordings were incorporated to quantify the evoked motor response to improve the initial design of this apparatus and ensure the accuracy and reliability of nerve stimulation. ***A***, The evoked motor activity was visualized through plots displaying raw intramuscular EMG from the gastrocnemius lateralis muscle (top) and torque recordings (bottom) for a given stimulation train at 1.0 mA. Plantar-flexion was verified in the torque recordings, consistent with gastrocnemius muscle activation. ***B***, Raw traces for both intramuscular EMG (top) and torque recordings (bottom) were aligned in time with a mean trace overlaid for a stimulation amplitude at 1.0 mA. The stimulation artifact was highlighted in gray for EMG recordings. ***C***, Mean traces of evoked EMG responses for corresponding stimulation amplitudes were aligned with respect to time. The stimulation artifact was highlighted in gray. ***D***, Mean traces of evoked torque recordings at the ankle joint for corresponding stimulation amplitudes were aligned with respect to time. ***E***, A fitted sigmoid curve demonstrated a strong relationship between the RMS values for EMG signals and peak torque measurements as stimulation amplitudes increased (*r* = 0.947; *p* < 0.005).

An acute rat sciatic nerve model was used to demonstrate the versatility of the hindlimb stabilization apparatus in measuring evoked motor activity, represented as intramuscular EMG from the gastrocnemius lateralis muscle and torque recordings. At a stimulation amplitude of 1.0 mA, a representative stimulation train from a representative subject (*n* = 1) was used to demonstrate recorded EMG and torque recordings (Fig. 8*A*). Peak torque within the stimulation train demonstrated a plantar-flexion response, which corresponds to gastrocnemius muscle activation. To further evaluate the relationship between evoked EMG and torque recordings, we extracted and aligned with respect to time the individual pulses from corresponding stimulation trains (Fig. 8*B*). The stimulation threshold for an evoked ankle displacement was visually observed at 0.5 mA. The evoked motor activity from stimulation amplitudes of 0.5–1.0 mA was recorded and aligned with respect to time, represented as mean traces (Fig. 8*C*,*D*). Maximum motor response of the gastrocnemius lateralis muscle was observed at 0.8 mA within the EMG recording (RMS, 1.99 ± 0.02 mV) and was not significantly different from responses at 0.9 and 1.0 mA (*p* < 0.005). This relationship was not observed within the torque measurements where peak response was first observed at 0.9 mA (40.8 ± 3.05 N-mm), suggesting additional muscle recruitment during ankle plantar-flexion (*p* < 0.005). Both functional metrics exhibited a strong correlation with each other (*r* = 0.947; *p* < 0.005; Fig. 8*E*). This suggests that recording noninvasive torque measurements around the ankle joint provides a quantifiable and reliable representation of muscle contraction and can be used as an alternative method compared with surgically invasive procedures.

## Discussion

Ankle torque measurements have been successfully demonstrated as a noninvasive tool in assessing evoked motor responses for chronic rat models (Jung et al., 2009; Howarth et al., 2020). However, existing designs lack comprehensive implementation instructions and may involve significant costs for additional hardware and expertise. The proposed hindlimb stabilization apparatus utilizes custom-made components constructed from 3D-printing and basic machining, offering a versatile and customizable solution for various experimental designs in evaluating evoked motor responses (Figs. 2–4). Most components (11/14) were 3D-printed using fused deposition modeling, enabling rapid prototyping at reduced manufacturing costs (Table 1-2). Additional hardware was sourced from a single manufacturer, simplifying raw material acquisition (Table 1-3). Other transducers may be used for implementation to reduce overall costs. Although a 6-DOF transducer was used, one torque axis was recorded for demonstration purposes. While calibration results demonstrated high accuracy (*r* = 0.999; *p* < 0.005; Fig. 5), the largest error occurred at 1 g with 322 and 5.25% error rates in the positive and negative direction, respectively (Table 1). Measurements below 1 N-mm may be questionable and should be omitted from future data analysis as these values approached the transducer's sensitivity limitations (0.39 N-mm; JR3).

Ankle torque measurements were the preferred noninvasive method when evaluating evoked motor responses as surface EMG recordings are highly susceptible to noise contamination, thus dampening the signal quality (De Luca et al., 2010; Qiu et al., 2015). Acute studies demonstrated that evoked ankle torque can be reliably recorded with respect to sciatic nerve stimulation at various amplitudes (*r* = 0.953; *p* < 0.005; Fig. 6). Peak torque was used as the main metric to evaluate sciatic nerve stimulation and demonstrated an increased response with respect to increased stimulation amplitudes. Regardless of operating experience, collected torque measurements from all users showed no observable differences, validating the versatility of the platform when proper procedures were followed (*p* < 0.005; Fig. 7). However, the expert operating experience may have biased the data collection as larger torque measurements were recorded, leading to a larger standard deviation (Table 7-1). Furthermore, the hindlimb stabilization apparatus successfully acquired torque measurements within the surgical environment, demonstrating compatibility with established anesthesia and rat vital monitoring setups.

A separate study was conducted to investigate the suitability of ankle torque measurements as a quantifiable metric for muscle contraction when compared with a more invasive and direct measurement, intramuscular EMG (Fig. 8). A novel implantable PNI, currently in development, was acutely evaluated for neuromodulation and electrophysiology recording capabilities. Sciatic nerve stimulation evoked ankle plantar-flexion, verified in intramuscular EMG (gastrocnemius lateralis) and torque recordings (Fig. 8). A strong relationship was observed between the peak torque and RMS values from EMG recordings with respect to increasing stimulation amplitudes (*r* = 0.947; *p* < 0.005; Fig. 8*E*). It is important to acknowledge that each method has its own limitations. However, using a variety of measurement techniques that complement one another can improve the study's rigor and reliability. Although a strong relationship was observed, motor responses from the gastrocnemius lateralis muscle appeared to reach a point of saturation at 0.8 mA (*p* < 0.005; Fig. 8*C*,*E*). However, larger responses were recorded in the torque measurements above this amplitude where saturation may have occurred 0.9 mA (*p* < 0.005; Fig. 8*D*,*E*). The sciatic nerve contains a bundle of nerves that innervates multiple muscles of the leg, suggesting recruitment of additional muscle groups during ankle flexion, such as the gastrocnemius medialis and/or soleus. Implantation of additional electrodes would be necessary to verify this observation as EMG recordings were limited to a single muscle group. However, this would result in an increase in invasiveness when evaluating the neuromuscular function. Although ankle torque measurements may lack the ability to selectively distinguish contracting muscle groups, the hindlimb stabilization apparatus provides a reliable and noninvasive alternative for evaluating in vivo applications of PNIs in development.

Although other studies have provided detailed methods for acquiring torque measurements, sufficient details on how to acquire or construct such equipment have been poorly documented, obstructing the replication of methods (Grill and Mortimer, 1996; Stieglitz et al., 2003; Ichihara et al., 2009; Iyer et al., 2016). In the study conducted by Iyer et al., a “custom-designed” foot plate and stabilization device incorporated a subcutaneously penetrating needle, used to secure the lower leg along the tibia and femur. In contrast, the hindlimb stabilization apparatus provides a complete noninvasive method to fully stabilize and secure the hindlimb while reducing errors in measuring ankle torque. Additionally, multiple components were designed to secure the hip, knee, and foot during motor contraction and may further be modified to meet specific experimental requirements such as accommodations of other anatomical structures, providing additional versatility while lowering implementation costs. CAD files of individual custom-made parts used in this study have been provided in an open repository. Additional hardware can be fitted into this design to address potential limb movement during or after stimulation and/or track positional errors beyond the initial placement.

Initially, this tool was designed to detect potential device failures for chronic applications of PNIs, such as lead migration, nerve damage, or electrode degradation, by measuring changes in torque values. While this method does not directly evaluate selectivity, additional degrees of freedom can be investigated to further explore selectivity, including inversion and eversion movements. It is important to note that this method cannot be used alone to reliably identify causes for electrode performance such as nerve damage, electrode failure, or migration. However, it may be used to indicate the presence of potential arising issues at the electrode–tissue interface and provide supporting evidence as part of a broader experimental design.

In this study, a hindlimb stabilization apparatus was developed and provides a customizable solution for consistent ankle joint measurements in rat models. This open-source, noninvasive platform enables reliable recording of evoked motor activity during nerve stimulation and can be used in various applications such as evaluating in vivo performance of neural interfaces. Utilizing 3D-printed components, this platform offers flexibility for additional design modifications to fit user needs.

## Synthesis

Reviewing Editor: Silvia Pagliardini, University of Alberta Decisions are customarily a result of the Reviewing Editor and the peer reviewers coming together and discussing their recommendations until a consensus is reached. When revisions are invited, a fact-based synthesis statement explaining their decision and outlining what is needed to prepare a revision will be listed below. The following reviewer(s) agreed to reveal their identity: Ana Hernandez.

This is an interesting paper that provides a detailed and affordable methodology for 3D-printing a platform allowing the assessment of neural interfaces for peripheral nerve stimulation. The methodology can enable the quick evaluation of novel peripheral nerve interfaces for stimulation through quantification of the sciatic nerve motor output of the rat model. While similar platforms have been reported in the past, albeit for larger animal models, this is the first well-documented 3D-printed platform that has been made available with open-source materials. This can improve standardization of reported outputs across labs for direct comparison of novel peripheral nerve interfaces, resulting in increased reproducibility and rigor for the field.

The paper provides detailed information on the design and parameters of the device. However, the novelty, uniqueness, and advantages of the device are not adequately highlighted in the article, and the measurement data appears limited in scope.

Main Comments

1. In the introduction, when discussing the advantages and disadvantages of different measurement methods, it would be valuable to specify how much more accurate ankle torque measurement is compared to surface EMG and video-based analyses. Are there specific references that provide such a comparison? It's important to acknowledge that some level of measurement error exists in each method. In the results section, the authors compare torque and EMG results, demonstrating their reliability. However, it would be beneficial to compare the results with kinematic data (e.g., joint angular velocity and acceleration) to see if similar trends emerge.

2. The paper briefly mentions related studies but fails to explain the specific superiority of the designed system compared to similar devices. While the paper mentions that the advantage of this design is its adjustability and customizability, it's worth noting that adjustable ankle torque systems have already been discussed in Lyer et al., 2016. Therefore, in the discussion section, it would be helpful for the authors to reiterate the unique advantages of their design beyond having specific design guidelines mentioned in existing literature. Also address the question of how this novel low-cost system compares to existing commercial systems in terms of cost-effectiveness and measurement accuracy. This discussion need not be limited to rat ankle torque measurement systems; there is ample research in human studies that can provide valuable insights.

3. Torque differences between the first and second in-vivo experiments: the torque observed for the first in-vivo experiment (Figure 6) shows approximately 20 N-mm evoked at 1 mA, but the same current amplitude for the second experiment resulted in a torque close to 40 N-mm (Figure 8). It is unclear whether these differences arose from variability in the torque measurements across experiments or the electrode characteristics (e.g., material, surface area, placement, etc.).

4. Validation of plantar flexion and dorsiflexion: it appears that all stimulation pulses resulted in plantar flexion of the ankle; however, calibration was carried out to assess both directions. It would be beneficial to attempt stimulation to evoke dorsiflexion (e.g., by targeting the common peroneal nerve) to fully validate 3D-printed platform for the assessment of both plantar flexion and dorsiflexion.

5. Limitation of goniometric range: how was it ensured that the placement of the hind limb into the foot pedal assembly did not limit the animal's goniometric range? This could potentially affect the peak torque.

6. Variability of the expert group: the variability of the normalized torque for the expert group is larger than the variability for the beginner and intermediate groups. One would expect that the variability would be smaller in the expert group. Please provide an interpretation of these observations.

7. The statistical analysis regarding the calculation of the Pearson's correlation coefficient is correct; however, the statistical methods performed to compare operator's user experience is not documented in this article.

Minor comments:

1. The paper mentions adjustability but does not specify the adjustable range. It only mentions that the device is suitable for rats weighing between 250-450g. Please provide specific adjustable length information.

2. Provide an overall picture of the device when the rat's lower limb is fixed.

3. The paper lacks information on the fixation of other body parts besides the tibia and ankle joint. The position of the rat's body could potentially affect torque generation.

4. Please explain what is meant by "normalized by the respective limb" in line 300. Is it referring to limb length?

5. In Table 1, please provide basic information about the rat subjects used, such as their ages, genders, and weights. And provide results from repeated measurements and include mean+-std to enhance the table's credibility. Given the large errors with small torque values, specify the minimum valid values achievable.

6. Please present results for other amplitude levels within the range of 0.5-5mA in Figure 6B and 6C. And clarify whether the data in Figure 6C and 6D are from multiple rats or just one.

7. In section 4.3 (Intra-operator variability), considering that each operator conducted measurements on only one rat, there may be bias concerns. The differences between the operators in Figure 7 appear more like individual differences and may not strongly support the conclusion of consistency.

8. In Figure 8C, it's difficult to see the EMG data at 1.0mA. In Figure 8E, using only 6 measurement samples for linear regression may not be convincing. Include more repeated measurements across different trials, legs, and individuals.5. Please include sex and strain of the animal carcasses measured to determine the appropriate dimensions for this 3D-printed platform.

9. It is not clear how the optimal angle for isometric contraction was established (line 154).

10. Please indicate how the theoretical force was calculated in the methods section.

11. Line 225 mentions hindlimb data that was excluded, please provide how many datapoints were excluded from the final analysis.

12. Line 270 is missing the table number (end of the sentence reads "as shown in Table .").

13. Figure 6: I could not understand what panel C represents, or why the torque is almost 0 at the beginning of the stimulation, maximum at around 50 seconds and decreasing again at the end of the stimulation. In addition, please indicate in the figure legend that the dose-response curve corresponds to peak torque.
