# Supplementary material for: An Open-Source 3D-Printed Hindlimb Stabilization Apparatus for Reliable Measurement of Stimulation-Evoked Ankle Flexion in Rat
Source: eNeuro. 2024 Mar 1;11(3):ENEURO.0305-23.2023. doi: 10.1523/ENEURO.0305-23.2023 (PMC10918511; doi:10.1523/ENEURO.0305-23.2023)
Supplement: Table 1-1 — Measurements from individual rat carcasses used in designing the hindlimb stabilization apparatus. * = Measurement was not taken for this subject. Download Table 1-1, DOC file. [file eneuro-11-ENEURO.0305-23.2023-s008.doc]

**Table 1-1.** **Measurements from individual rat carcasses used in designing the hindlimb stabilization apparatus.** * = Measurement was not taken for this subject.

|  | **Body** | | **Lower Leg** | **Ankle** | | **Foot** | | **Center of Ankle Joint** | |
| --- | --- | --- | --- | --- | --- | --- | --- | --- | --- |
| Subject | Length (mm) | Width (mm) | Length (mm) | Width (mm) | Length (mm) | Length (mm) | Anterior Width (mm) | From Sole (mm) | From Heel (mm) |
| 1 | 22.0 | 8.00 | 45.0 | 6.00 | 6.00 | 45.0 | 13.0 | 5.00 | 7.00 |
| 2 | 18.0 | 6.50 | 35.0 | 5.50 | 7.00 | 35.0 | 12.0 | 5.00 | 4.00 |
| 3 | 21.0 | 8.50 | 50.0 | 6.00 | 8.00 | 49.0 | 11.0 | 7.00 | 5.00 |
| 4 | 24.0 | 9.50 | 35.0 | 5.00 | 8.00 | 45.0 | 9.00 | 5.00 | 6.00 |
| 5 | 20.0 | 8.00 | 35.0 | 5.00 | 7.00 | 47.0 | 15.0 | 5.00 | 5.00 |
| 6 | 26.0 | 10.0 | 38.0 | 6.00 | 7.00 | 48.0 | 15.0 | 6.00 | 5.00 |
| 7 | * | * | * | 5.00 | 8.00 | 45.0 | 15.0 | * | * |
| 8 | 19.0 | 5.00 | 35.0 | * | * | * | * | 6.00 | 7.00 |
| 9 | 16.5 | 6.50 | 37.0 | * | * | * | * | 5.00 | 5.00 |
| Mean | 20.8 | 7.75 | 38.8 | 5.50 | 7.29 | 44.9 | 12.9 | 5.50 | 5.50 |
| STD | 2.94 | 1.56 | 5.31 | 0.46 | 0.70 | 4.29 | 2.17 | 0.71 | 1.00 |
