# Supplementary material for: An Open-Source 3D-Printed Hindlimb Stabilization Apparatus for Reliable Measurement of Stimulation-Evoked Ankle Flexion in Rat
Source: eNeuro. 2024 Mar 1;11(3):ENEURO.0305-23.2023. doi: 10.1523/ENEURO.0305-23.2023 (PMC10918511; doi:10.1523/ENEURO.0305-23.2023)
Supplement: Table 1-2 — Printing specifications for 3D-printed components. Infill (%), print time (hours, minutes), and unit mass (g) were calculated using the Ultimaker Cura software. All components were printed using PLA. * = Unit price calculated at $0.15 (USD) per gram. Download Table 1-2, DOC file. [file eneuro-11-ENEURO.0305-23.2023-s009.doc]

**Table 1-2. Printing specifications for 3D-printed components**. Infill (%), print time (hours, minutes), and unit mass (g) were calculated using the Ultimaker Cura software. All components were printed using PLA. * = Unit price calculated at $0.15 (USD) per gram.

| **Printed Component** | **Qty** | **Infill (%)** | **Print Time** | **Unit Mass (g)** | **Unit Price*** | **Total Price** |
| --- | --- | --- | --- | --- | --- | --- |
| Foot pedal | 1 | 80 | 2 hr 24 min | 12 | $1.80 | $1.80 |
| Flange mount shaft collar | 1 | 20 | 51 min | 4 | $0.60 | $0.60 |
| Railing mount cap | 1 | 20 | 2 hr 12 min | 10 | $1.50 | $1.50 |
| Railing mount | 1 | 50 | 15 hr 44 min | 90 | $13.50 | $13.15 |
| Base attachment | 1 | 50 | 12 hr 3 min | 61 | $9.15 | $9.15 |
| Body stabilizer | 1 | 50 | 7 hr 34 min | 36 | $5.40 | $5.40 |
| Knee Clamp | 1 | 50 | 2 hr 55 min | 12 | $1.80 | $1.80 |
| Height adjustment plate | 1 | 20 | 9 min | 1 | $0.15 | $0.15 |
| Mount for knee clamp railing | 4 | 20 | 25 min | 2 | $0.30 | $1.20 |
| Left foot pedal railing support | 1 | 50 | 4 hr 7 min | 21 | $3.15 | $3.15 |
| Right foot pedal railing support | 1 | 50 | 3 hr 43 min | 18 | $2.70 | $2.70 |
