# Supplementary material for: An Open-Source 3D-Printed Hindlimb Stabilization Apparatus for Reliable Measurement of Stimulation-Evoked Ankle Flexion in Rat
Source: eNeuro. 2024 Mar 1;11(3):ENEURO.0305-23.2023. doi: 10.1523/ENEURO.0305-23.2023 (PMC10918511; doi:10.1523/ENEURO.0305-23.2023)
Supplement: Table 1-3 — Bill of materials for additional hardware used in design implementation. Cost is shown at time of lookup (circa 2021). * = Unit cost covers the quantity required. Download Table 1-3, DOC file. [file eneuro-11-ENEURO.0305-23.2023-s010.doc]

**Table 1-3. Bill of materials for additional hardware used in design implementation**. Cost is shown at time of lookup (circa 2021). * = Unit cost covers the quantity required.

| **Item** | **Description** | **Source** | **Part Number** | **Qty** | **Additional Machining** | **Unit Cost*** |
| --- | --- | --- | --- | --- | --- | --- |
| Multipurpose 304 SS Rod- 10mm diameter | Shaft piece for transducer | McMaster | 1272T41 | 1 | Vertical bandsaw, mill | $13.33 |
| Multipurpose 6061 Aluminum ½” Thick | Transducer mount | McMaster | 8975K477 | 1 | Vertical bandsaw, mill | $7.73 |
| Long Guide Rail for 63 mm Wide Carriage | Railing for sleeve bearing carriage | McMaster | 9867K13 | 1 | Vertical bandsaw | $27.50 |
| Locking sleeve bearing carriage for 20mm Wide Rail | Sleeve bearing carriage | McMaster | 3249K3 | 1 | - | $61.42 |
| 20E12A4-I25-EF-10N Transducer | Torque transducer | JR3 | 20E12A4 | 1 | - | $9930.54 |
| Multipurpose Neoprene Rubber Sheet | Grip surface for knee clamp | McMaster | 1374N23 | 1 | - | $21.09 |
| Clear Cast Acrylic Sheet | Acrylic base | McMaster | 8560K269 | 1 | CNC Router | $145.08 |
| Rubber foot | Base stabilization | McMaster | 53535A45 | 4 | - | $11.95 |
| T-Slotted Framing Low Profile 1-Slot Single Rail | Base railing for foot pedal | McMaster | 6812N15 | 1 | Vertical bandsaw | $11.12 |
| Miniature T-Slotted Framing Single Rail | Base railing for knee clamp | McMaster | 1959N1 | 1 | Vertical bandsaw | $4.95 |
| Brass Heat-Set Inserts for Plastic, M4 | Threads for knee clamp, railing mount | McMaster | 92259A150 | 8 | - | $9.84 |
| Brass Heat-Set Inserts for Plastic, M5 | Threads for carriage railing | McMaster |  | 1 | - |  |
| Brass Heat-Set Inserts for Plastic, M6 | Threads for foot pedal | McMaster | 94459A190 | 1 | - | $14.82 |
| 18-8 SS Socket Head Screw, M4 x 0.7mm | Fixation for shaft collar, railing cap, knee clamp height | McMaster | 91292A116 | 8 | - | $7.55 |
| 18-8 SS Hex Drive Flat Head Screw, M4 x 0.7mm | Fixation for base attachment, acrylic base railing attachments | McMaster | 92125A186 | 10 | - | $6.12 |
| High-Strength A286 SS Socket Head Screw, M4 x 0.7mm | Knee clamp adjustability | McMaster | 90035A129 | 1 | - | $4.14 |
| 18-8 SS Socket Head Screw, M5 x 0.8mm | Fixation for transducer mount, carriage railing | McMaster | 91292A125 | 6 | - | $10.93 |
| 18-8 SS Hex Drive Flat Head Screw, M6 x 1mm | Foot pedal set screw | McMaster | 9129A134 | 1 | - | $8.52 |
| T-Slotted Framing Drop-in Hammer Nut with Button Head | T-nut for foot pedal base railing | McMaster | 5537T73 | 4 | - | $2.52 |
| Button Head Cap Screw for Miniature T-Slotted Framing | Fixation for knee clamp base railing | McMaster | 1959N39 | 4 | - | $8.12 |
| Nut Plate for Miniature T-Slotted Framing | T-nut for knee clamp base railing | McMaster | 1959N37 | 6 | - | $8.12 |
