# Supplementary figures and images for: An Open-Source 3D-Printed Hindlimb Stabilization Apparatus for Reliable Measurement of Stimulation-Evoked Ankle Flexion in Rat
Source: eNeuro. 2024 Mar 1;11(3):ENEURO.0305-23.2023. doi: 10.1523/ENEURO.0305-23.2023 (PMC10918511; doi:10.1523/ENEURO.0305-23.2023)

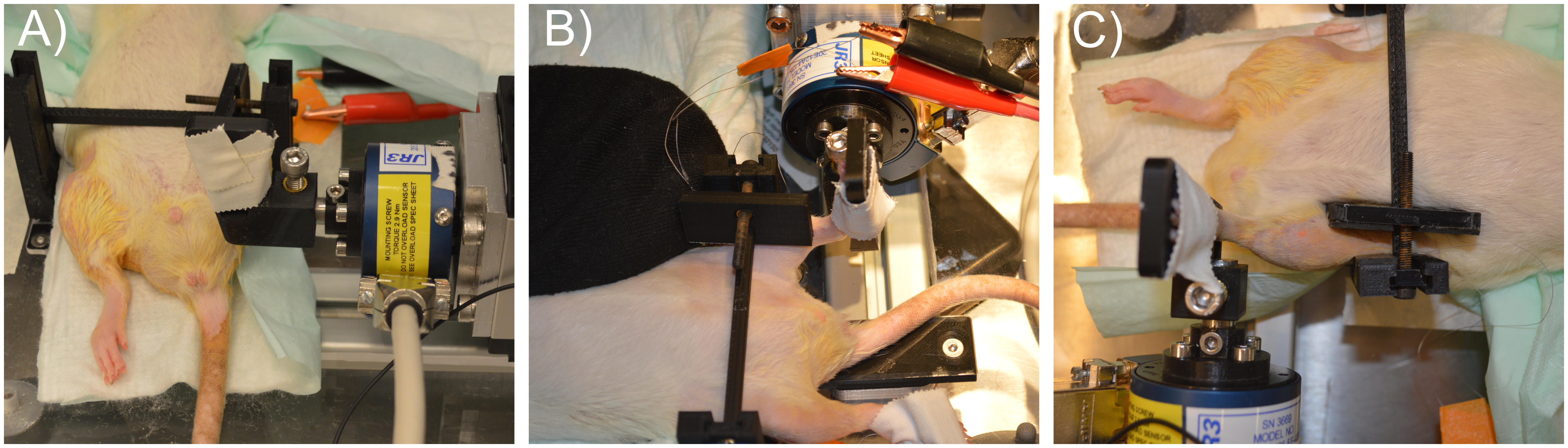

Supplement: Figure 1-1 — Example placement of the rat's left hindlimb. A, B, C) Additional views were provided to illustrate proper securement of the hindlimb after proper adjustment of the knee-locking and foot pedal assemblies. Download Figure 1-1, TIF file. [file eneuro-11-ENEURO.0305-23.2023-s002.tif]

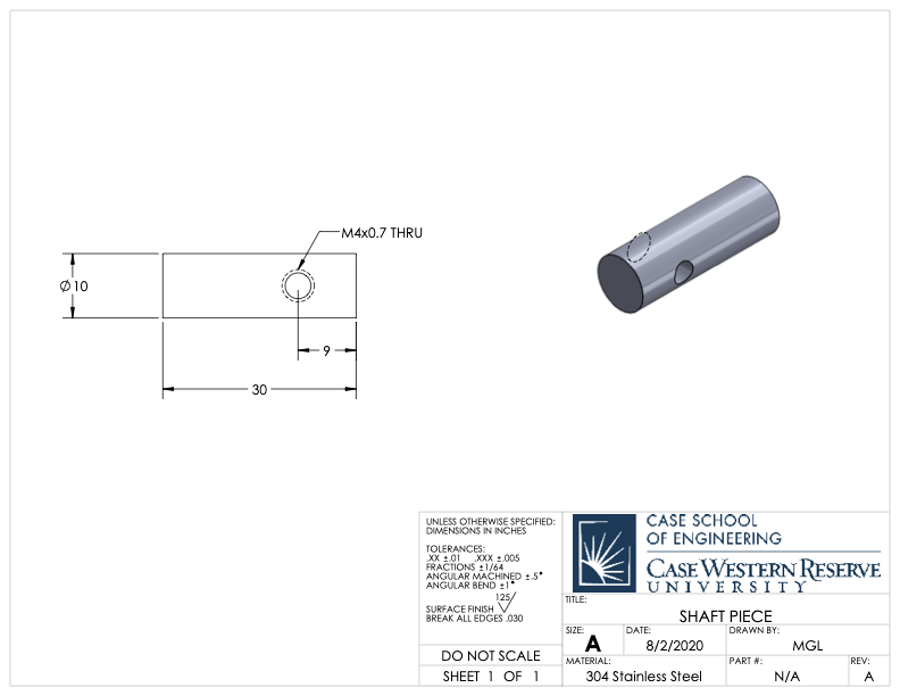

Supplement: Figure 4-1 — Dimensions (inches) for the cut stainless-steel shaft used to connect and align the torque transducer to the foot pedal. Download Figure 4-1, TIF file. [file eneuro-11-ENEURO.0305-23.2023-s003.tif]

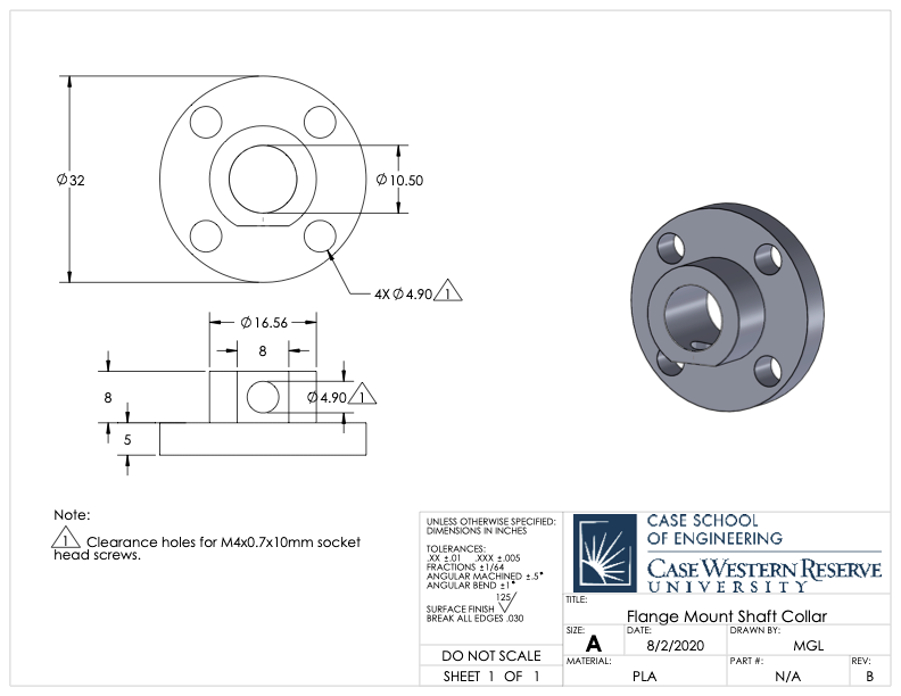

Supplement: Figure 4-2 — Dimensions (inches) for flanged-mount shaft collar used to couple the foot pedal's shaft to the torque transducer. Download Figure 4-2, TIF file. [file eneuro-11-ENEURO.0305-23.2023-s004.tif]

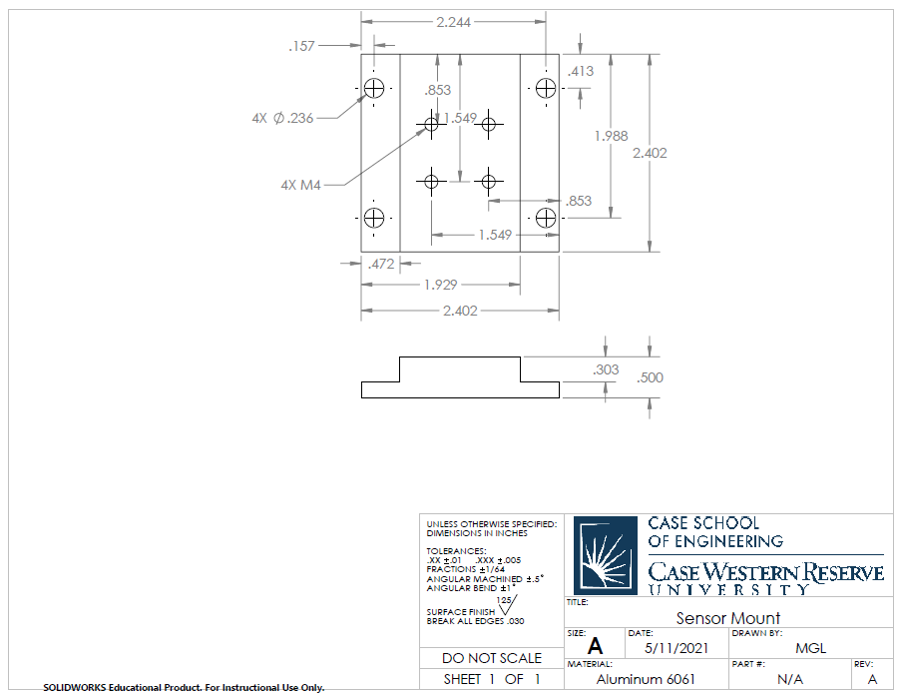

Supplement: Figure 4-3 — Dimensions (inches) for machined mount used for the torque transducer in the foot pedal assembly. Download Figure 4-3, TIF file. [file eneuro-11-ENEURO.0305-23.2023-s005.tif]

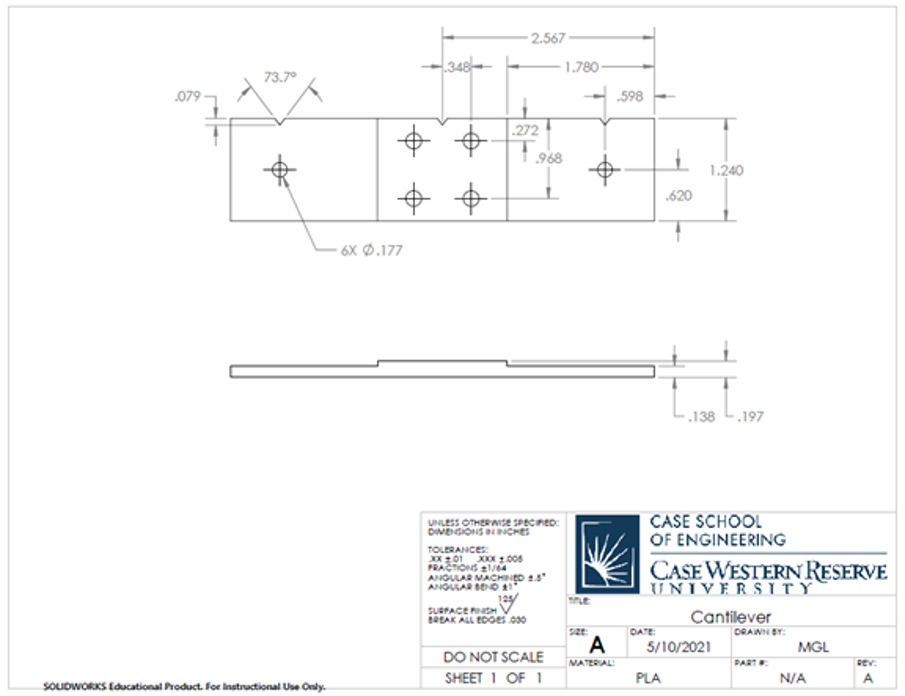

Supplement: Figure 5-1 — Cantilever dimensions (inches) for calibration of the torque transducer. Download Figure 5-1, TIF file. [file eneuro-11-ENEURO.0305-23.2023-s006.tif]

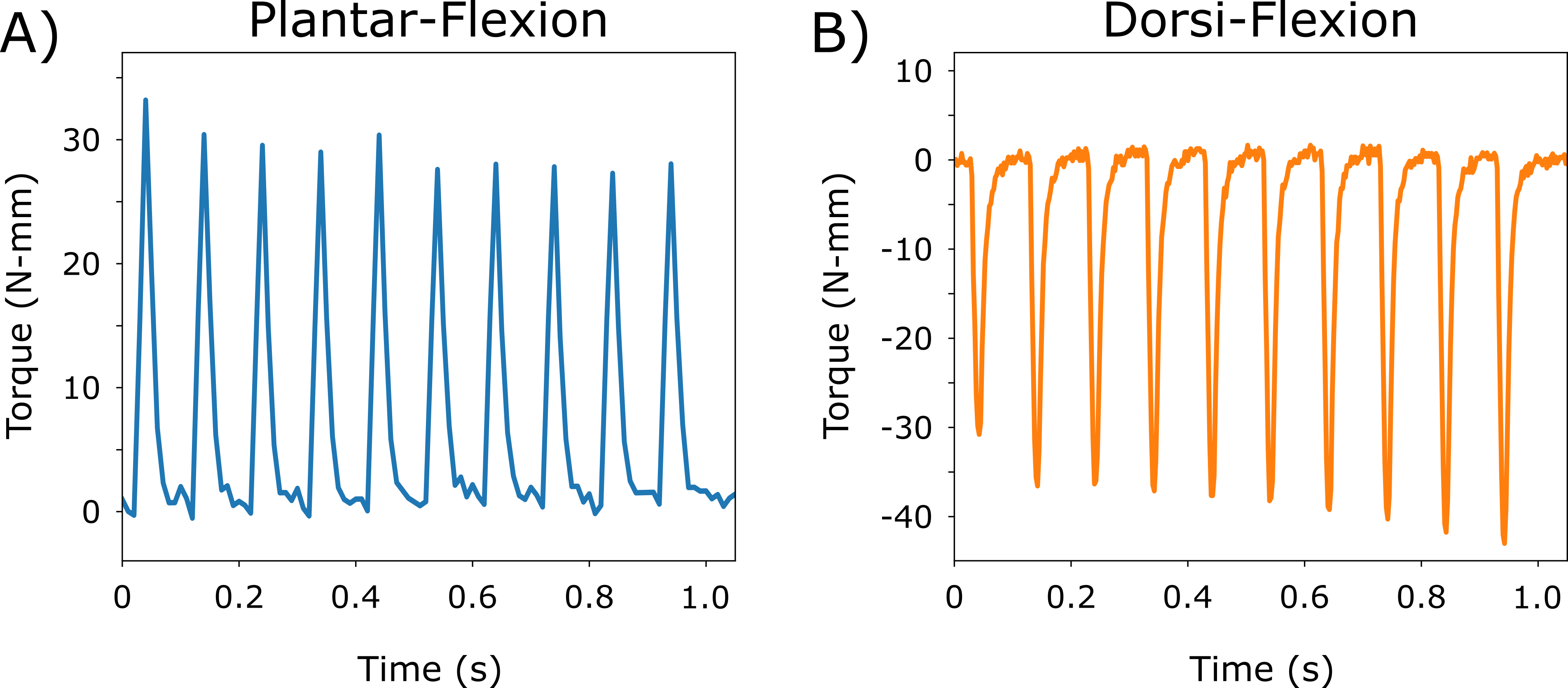

Supplement: Figure 6-1 — Example of recorded torque measurements for A) plantar- and B) dorsi-flexion. Respective ankle flexions were evoked with the following stimulation parameters: 1.0mA, 10Hz frequency, 100μs pulse width, and 25μs inter-pulse delay. Download Figure 6-1, TIF file. [file eneuro-11-ENEURO.0305-23.2023-s007.tif]
