# Supplementary material for: An Open-Source 3D-Printed Hindlimb Stabilization Apparatus for Reliable Measurement of Stimulation-Evoked Ankle Flexion in Rat
Source: eNeuro. 2024 Mar 1;11(3):ENEURO.0305-23.2023. doi: 10.1523/ENEURO.0305-23.2023 (PMC10918511; doi:10.1523/ENEURO.0305-23.2023)
Supplement: Table 7-1 — Individual normalized torque recordings collected by respective operators. Download Table 7-1, DOC file. [file eneuro-11-ENEURO.0305-23.2023-s011.doc]

**Table 7-1. Individual normalized torque recordings collected by respective operators**.

| *Subject 1* | | | | | | |
| --- | --- | --- | --- | --- | --- | --- |
| ***Operator*** | ***Limb Side*** | ***Normalized Torque*** | | | ***Mean*** | ***Std*** |
| Beginner | Left | 0.45 | 0.47 | 0.49 | 0.47 | 0.02 |
|  | Right | 0.70 | 0.94 | 1.13 | 0.93 | 0.18 |
| Intermediate | Left | 0.86 | 1.00 | 1.13 | 1.00 | 0.11 |
|  | Right | 0.29 | 0.44 | 0.54 | 0.42 | 0.10 |
| Expert | Left | 1.50 | 1.66 | 1.79 | 1.65 | 0.12 |
|  | Right | 1.30 | 1.56 | 1.74 | 1.53 | 0.18 |
| *Subject 2* | | | | | | |
| ***Operator*** | ***Limb Side*** | ***Normalized Torque*** | | | ***Mean*** | ***Std*** |
| Beginner | Left | 0.89 | 1.13 | 1.31 | 1.11 | 0.17 |
|  | Right | 0.54 | 0.71 | 0.80 | 0.68 | 0.11 |
| Intermediate | Left | 1.40 | 1.44 | 1.50 | 1.45 | 0.04 |
|  | Right | 0.40 | 0.50 | 0.57 | 0.49 | 0.07 |
| Expert | Left | 1.43 | 1.84 | 2.20 | 1.82 | 0.31 |
|  | Right | 0.33 | 0.46 | 0.54 | 0.44 | 0.08 |
| *Subject 3* | | | | | | |
| ***Operator*** | ***Limb Side*** | ***Normalized Torque*** | | | ***Mean*** | ***Std*** |
| Beginner | Left | 1.33 | 1.08 | 1.26 | 1.23 | 0.11 |
|  | Right | 0.81 | 1.19 | 1.46 | 1.15 | 0.27 |
| Intermediate | Left | 1.13 | 1.13 | 1.11 | 1.12 | 0.01 |
|  | Right | 0.57 | 0.69 | 0.78 | 0.68 | 0.09 |
| Expert | Left | 0.90 | 1.21 | 1.39 | 1.17 | 0.21 |
|  | Right | 0.50 | 0.65 | 0.81 | 0.65 | 0.13 |
| *Total* | | | | | | |
| ***Operator*** | ***Mean*** | ***Std*** |  | ***Median*** | ***Min*** | ***Max*** |
| Beginner | 0.93 | 0.31 |  | 0.92 | 0.45 | 1.46 |
| Intermediate | 0.86 | 0.37 |  | 0.82 | 0.29 | 1.50 |
| Expert | 1.21 | 0.55 |  | 1.35 | 0.33 | 2.20 |
